# Contextual Support for Less Salient Homophones and Pun Humor Appreciation: Evidence From Eye Movements in Reading Chinese Homophone Puns

**DOI:** 10.3389/fpsyg.2022.875479

**Published:** 2022-05-11

**Authors:** Wei Zheng, Xiaolu Wang

**Affiliations:** ^1^School of Foreign Languages, China Three Gorges University, Yichang, China; ^2^School of Foreign Languages, Zhejiang University City College, Hangzhou, China; ^3^School of International Studies, Zhejiang University, Hangzhou, China; ^4^School of Humanities and Communication Arts, Western Sydney University, Penrith, NSW, Australia

**Keywords:** contextual information, Chinese homophone puns, less salient homophone, humor appreciation, eye movements

## Abstract

Punning is an important means of creating humorous effects by intentionally exploiting semantic ambiguity. Previous psycholinguistic research on puns has mainly focused on the process of meaning retrieval in homograph puns, while it is still not entirely clear how readers dynamically utilize contextual information to understand homophone puns. In the current investigation, 68 native Chinese participants were recruited to read three types of experimental sentences while their eye movements were recorded: (1) the homophone-pun sentences where the less salient homophone was visually presented, (2) the homophone-salient sentences where the salient homophone was used, and (3) the homophone-error sentences where the critical context noun in the homophone puns was replaced with an unrelated word. Humor rating results of the homophone puns and the homophone-salient sentences demonstrated that the less salient homophones rather than the salient ones elicited much larger humor responses when presented visually in the same potential pun context. In addition, the reverse fixation pattern in the homophone area and the spill-over region also suggested that meanings of the salient homophones were more recoverable even when not presented visually. Statistical analyses of the homophone puns and the homophone-error sentences showed that the semantic relatedness between the critical context noun and the less salient homophone could significantly predict the humor rating scores of Chinese readers. Taken together, less salient homophones need to receive more contextual support to balance out the advantages of salient homophones before generating a humorous pun interpretation.

## Introduction

Punning is an essential type of verbal humor allowing for two interpretations simultaneously. Based on the critical ambiguous word (the pun word), puns can be broadly divided into two types: homograph puns, where the two meanings derive from one homograph (e.g., *A happy life depends on a liver*); homophone puns, where the two meanings come from a homophone pair (e.g., *A bicycle can stand on its own because it is two-tired*). Although puns may generate certain ambiguity or uncertainty, which is detrimental to general communication, most people seem to enjoy the amusement that usually comes with them. For example, one classic Chinese pun goes like: *东边日出西边雨, 道是无晴却有晴* (*The rain comes along with the sunlight; it is not yet sunny, but she thinks of her Sonny*). Composed centuries ago, this poem sentence is still appreciated by most modern Chinese readers for its ingenious punning (*有晴/有情 sunny/affectionate*).

Due to the wide use of puns, a sizeable psycholinguistic literature has accumulated on its comprehension process in recent decades. Intuitively, successful access to the two meanings of the pun word is a prerequisite for pun comprehension, without which no juxtaposition of two interpretations is possible. As a result, most current investigations have focused on the meaning-access process during pun comprehension. Although different terms may have been used, one consistent finding in recent pun research is the so-called salience effect; namely, the salient meaning of a pun word is accessed faster than the other related but less salient meaning (e.g., Zheng et al., 2020), in line with the graded salience hypothesis (Giora, 1997). This theory adopts a modular view of linguistic processing (Fodor, 1983). It claims that the salient (more frequent, familiar, conventional, or prototypical) meaning associated with a linguistic expression (written form or sound) will be invariantly accessed faster than the less salient ones regardless of contextual support. Nevertheless, this hypothesis does not deny the influence of context but claims that the lexical information and contextual information are processed through two parallel systems. Specifically, the former is governed by a modular lexical mechanism that only utilizes information inside the mental lexicon. In contrast, the latter is processed by a prediction-based general system that can incorporate both linguistic input and world knowledge (Peleg et al., 2001).

In their seminal event-related potential (ERP) study on the temporal dynamics of pun comprehension, Coulson and Severens (2007) recorded the ERPs while their participants were listening to homograph puns (e.g., *During branding, cowboys have sore calves*). Three different types of probes were visually presented in either the left or right visual field of the participants (the divided visual field paradigm), including probes related to the salient meaning (e.g., *cow*), probes related to the less salient meaning (e.g., *leg*), and unrelated probes (e.g., *stroke*). It was found that both the semantically-related probes elicited smaller N400 amplitudes^1^ than the unrelated controls in the left hemisphere when presented immediately after the pun word (e.g., *calves*) offset, indicating that these meanings had already been retrieved. But only the probes related to the salient meaning yielded a similar priming effect in the right hemisphere. In contrast, such asymmetry disappeared when the probes were presented 500 ms after the pun word offset. Namely, both semantically-related probes became equally available in both hemispheres 500 ms after the pun word was fully heard. These results show that the two meanings of the pun word are not accessed simultaneously; rather, the salient meaning will be available first (at least in the left hemisphere).

The salience effect during pun comprehension was further supported by many following studies using different paradigms, such as behavioral study (Mchugh and Buchanan, 2016; Koleva et al., 2019) and eye-tracking study (Zheng et al., 2020). For example, Zheng et al. (2020) used a visual world paradigm to investigate how the two meanings of the pun word are accessed temporally. In their experiment, the participants listened to Chinese homograph and homophone puns while looking at a visual display of four printed words: a phonological competitor (words sharing the first syllable with the pun word), two semantic competitors (words related to both meanings of the pun word), and an unrelated control. According to the results, participants fixated significantly more on the semantic competitors related to the salient meaning after the pun word was fully heard. However, this advantage disappeared 200 ms later when both semantic competitors received more fixations than the phonological competitor and the unrelated word. As a result, the authors claimed that their findings exemplified the salient meaning advantages proposed by the graded salience hypothesis.

Although the salience effect in pun comprehension has been increasingly supported by recent research, one problem with this conclusion is that most of these studies are based on homograph puns. Therefore, it is still unclear whether the same effect can be observed during homophone comprehension. It is reasonable to raise such concern because homograph puns and homophone puns are quite different, especially in terms of reading. When reading a homograph pun, readers can retrieve the two meanings of the pun word based on the same orthographic and phonological information. In contrast, homophone puns employ a pair of orthographically-different homophones, and usually, only one of them is presented visually. Consequently, readers can only acquire one meaning through the presented homophone while relying heavily on the shared phonology and context for the other meaning. Although some homophone puns were used in the study of Zheng et al. (2020), their materials were presented to the participants *via* recordings; hence the differences between the two types of puns were largely masked in that the participants had to rely on phonological information for both types of puns.

The current study then further examines the salience effect in reading homophone puns. In particular, we answer the question: which of the homophone-pair to present is more likely to result in a pun interpretation (Zheng et al., 2020)? According to the graded salience hypothesis, the salient meaning associated with a linguistic form or sound is more recoverable even when the context is not in its favor. Therefore, it predicts that visual presentation of the less salient homophone rather than the salient homophone is more likely to result in a pun interpretation. If this prediction is accurate, the salience effect can be a robust effect during pun comprehension in general. To date, there seems to be no study that has directly investigated the salience effect in reading homophone puns. As a result, the current study is significant because it can give us a complete picture of the salience effect during pun comprehension.

Another research question of the current study is what makes readers consider certain puns as funnier than others? In a pioneering eye-tracking study on reading homophone puns, Jared and Bainbridge (2017) compared English homophone puns (e.g., *The butcher was very glad that we could meat up*) with their homophone-error controls (e.g., *The lawyer was very glad we could meat up*) where the presented homophones (e.g., *meat*) were not supported. Although no difference was found in either the first fixation duration or gaze duration on the homophones, the total reading time did, suggesting the critical context noun (e.g., *butcher*) facilitated its integration in the homophone-pun condition. Interestingly, they found that the semantic relatedness between the presented homophone (e.g., *meat*) and the critical context word (e.g., *butcher*) was a reliable predictor for the funniness rating scores toward the homophone puns. This finding is significant in that it has identified, probably for the first time, a quantifiable factor to predict readers’ response when reading homophone puns.

However, whether this measure can also predict humor ratings toward homophone puns in other scripts, such as Chinese, still waits for further investigation. Compared with alphabetic languages (e.g., English), the logographic writing system of Chinese is more opaque; namely, the relationship between the orthographic form and its pronunciation is weaker. As a result, it is very common to find Chinese homophone mates with little visual similarities (e.g., *男伴-难办*/nan_2_ban_4_/), in contrast to English homophone pairs (e.g., *meat-meet*/mi:t/). Besides, previous research suggests that skilled Chinese readers, such as native Chinese college students, can retrieve semantic information directly, bypassing the phonological route (Zhou et al., 2018). In light of such topological differences, the current study also examines the reliability of this measure for Chinese homophone puns.

In the present eye-tracking experiment, the participants were required to read three different types of sentences and rate how humorous they considered each sentence. To answer the first research question, we compared the humor rating scores between homophone-salient and homophone-pun sentences: the condition with higher scores could reflect that readers were more likely to consider it a pun. This question is not trivial because, theoretically speaking, either the salient or the less salient homophone could lead to a pun interpretation when presented in the same potential pun context, with their unpresented homophone mates retrieved through shared phonology. In addition, eye-tracking measures, such as the regression proportion, could shed light on the recoverability of the salient and less salient homophones when not presented. Specifically, the unpresented homophones whose meanings are more recoverable should result in fewer regressions to the critical context noun region since it should rely less on contextual support (Rayner et al., 1998). To answer the second research question, we compared the rating scores for the homophone-pun and homophone-error sentences. Linear mixed-effect models were used to test whether the semantic relatedness between the less salient homophone and the context word could serve as a predictor for the humor rating scores of Chinese readers. Answers to these questions are not only beneficial for understanding the reading process of homophone puns but also can help to shed more light on the unique features of pun context.

## Materials and Methods

### Participants

A group of 68 native Chinese speakers (22 males and 46 females, mean age = 21.7, *SD* = 2.5) participated in the experiment. They were students studying at a key university in China and had normal or corrected-to-normal vision. Participants were recruited through the campus forum and were paid a small amount after the experiment. The experiment received approval from the research ethics board of the university.

### Materials

A total of 72 sentence triads were created for the experimental materials (See Table 1 for a sample of the three sentence types). In the beginning, 80 potential homophone-pun sentences were collected from Chinese newspaper headlines and the internet. These sentences were separated with a comma into two sections: the topic section and the homophone section. The topic section provides information about the general topic of the whole sentence, including a critical context noun that supports the less salient homophone; the homophone section completes the entire sentence with the less salient homophone whose salient counterpart is also semantically compatible with the overall sentence context. The sentences were slightly modified so that the homophones were not the last word to avoid potential wrap-up effects. The saliency (association strength) between the two homophone mates (e.g., *男题-难题*) and their shared phonology (e.g., /nan_2_ ti_2_/) was assessed by a group of 30 students who did not participate in the experiment. They were invited to write down the first word they could think of based on Chinese *pinyin*. A final 72 homophone-pun sentences were selected, in which the unpresented homophone mate was rated as salient by more than 90% of the participants.

**Table 1:** Sample sentences and the setting up of different region of interests (ROIs).

Condition	Example sentences	Critical context noun (ROI_1_)	Homophone (ROI_2_)	Spill-over region (ROI_3_)
Homophone-pun	陈氏男科医院, 您的男题我们解决。(Chen’s andrology hospital, your male problems we solve.)	男科 (Andrology)	男题 (Male problems)	我们解决 (We solve)
Homophone-salient	陈氏男科医院, 您的难题我们解决。(Chen’s andrology hospital, your difficulties we solve.)	男科 (Andrology)	难题 (Difficulties)	我们解决 (We solve)
Homophone-error	陈氏牙科医院, 您的男题我们解决。(Chen’s dental hospital, your male problems we solve.)	牙科 (Dental)	男题 (Male problems)	我们解决 (We solve)

English translations were given in parentheses.

Two control conditions were prepared for comparison: the homophone-salient sentence and the homophone-error sentence. The homophone-salient sentences were designed by only replacing the homophone in the homophone-pun sentences with its salient homophone mate, minimizing possible differences caused by the stimulus characteristics. In addition, the homophone-error sentences were created by manipulating the semantic relatedness between the critical context word and the pun word. To achieve this, the critical context noun (e.g., *男科*: andrology department), which supported the less salient homophone (e.g., *男题*: male problem), was replaced with an unrelated noun (e.g., *牙科*: dental department) matched in both word frequency and character strokes (*p*s > 0.10). Another group of 30 students, who did not participate in the experiment, rated the semantic relatedness between the critical context noun and the presented homophones in the three conditions on a five-point Likert scale, with 1 for highly unrelated and 5 for highly related. According to the rating results, the critical context noun in the homophone-pun condition was rated significantly more related to the less salient homophone than in both the homophone-error and homophone-salient conditions (*p*s < 0.001). See Table 2 for the properties of the context noun.

**Table 2:** Properties of the critical context noun used in the three sentence conditions.

Sentence type	Mean word frequency	Mean stroke number	Semantic relatedness
Homophone-pun	20.54	16.93	3.69
Homophone-error	22.03	17.18	1.95
Homophone-salient	20.54	16.93	2.35

“Semantic Relatedness” refers to the extent to which the critical context noun is semantically related to the presented homophone. Word frequency is measured in occurrences per million.

Three counterbalanced lists were generated based on the 72 sentence triads using a Latin square design so that each participant would only see one sentence from each triad. Before the experiment, another group of 45 students was recruited and randomly assigned to one of the three lists to rate the readability of the three sentence types. According to the results, the homophone-salient sentences were rated equally understandable (*M* = 3.81, *SD* = 0.37) as the homophone-pun sentences (*M* = 3.91, *SD* = 0.44, *p* > 0.10), while the homophone-error sentences were rated significantly more difficult to understand than the homophone-pun sentences (*p* < 0.001). However, the rating scores (*M* = 3.44, *SD* = 0.45) indicated that the homophone-error sentences were still understandable to some extent. In addition, 48 filler sentences chosen from similar sources were added to each list. As a result, every participant read 120 sentences during the eye-tracking experiment (24 homophone-pun sentences, 24 homophone-error sentences, 24 homophone-salient sentences, and 48 filler sentences).

### Apparatus

Eye movements were tracked with the SR Research Eyelink 1000 plus system, with a sampling rate of 1,000 Hz. Eye movement data were collected from the right eye only. The experiment was carried out on a 19-inch monitor (Dell P1917S) with a refresh rate of 75 Hz and a screen resolution of 1024 × 768 pixels. A chin-rest with forehead support was used for all participants to minimize head movements during the experiment.

### Procedures

After the participants entered the lab, they were briefly introduced to how the eye tracker works as well as the instructions for the experiment. The participants were seated 72 cm from the video monitor. Since all the trial sentences were presented in only one line, a three-point horizontal calibration and validation procedure was implemented during the experiment. The average validation error was less than 0.5° of visual angle.

At the beginning of each trial, a cross sign appeared on the left side of the screen. If no fixation were detected within the 1° of visual angle from the cross center within 5 s, the calibration procedure would be initiated again. Once a constant fixation was detected at the cross for 500 ms, a sentence would be presented with its first character replacing the cross. The participants needed to press the space bar on the keyboard once they finished reading the sentence. The sentence then disappeared and was replaced by a screen of a five-point Likert scale, in which the participants needed to rate the funniness of the previous sentence with a mouse. Once they clicked on any of the five scores, a yes/no comprehension question was given after 25% of the sentences, which was designed to ensure that the participants paid attention to the meaning of the sentence (Li et al., 2019).

**Figure 1:**
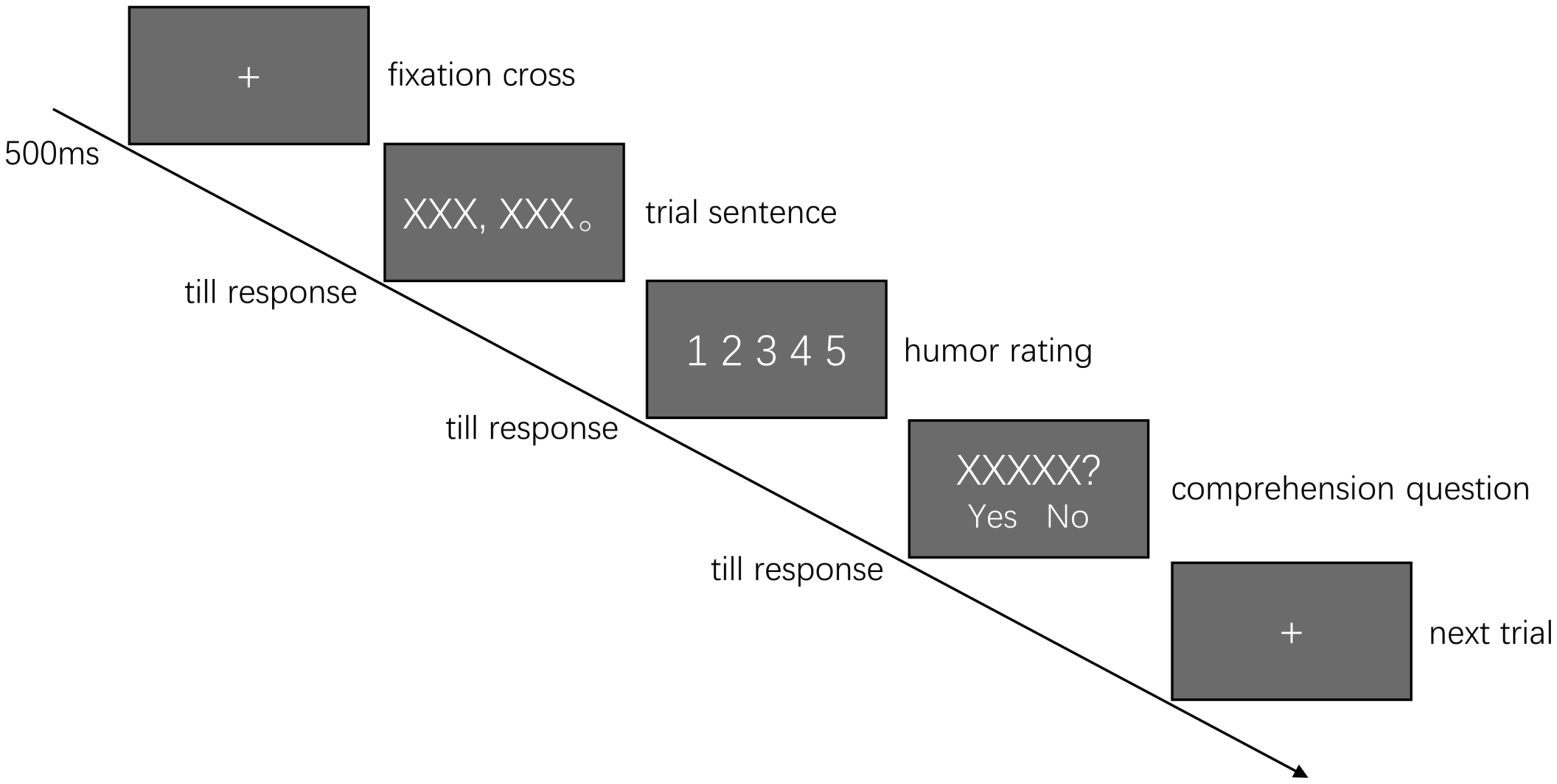
An illustration of the experiment paradigm.

Each participant was given six practice trials to familiarize themselves with the experimental procedure. Then, the 120 experimental trials were pseudo-randomly separated into four 30-trial blocks, with each sentence type appearing no more than twice successively. The participants took a short break after finishing each block. For each participant, the entire experimental session lasted for approximately 40 minutes. The experiment procedure is illustrated in Figure 1.

## Results

The mean accuracy of the comprehension questions was 91.8% (*SD* = 4.4), suggesting that the participants, in general, had understood the experimental sentences. Data from one participant were dropped for further data analysis due to low comprehension accuracy (79%).

### Sentence-Level Analysis

#### Humor Ratings

**Figure 2:**
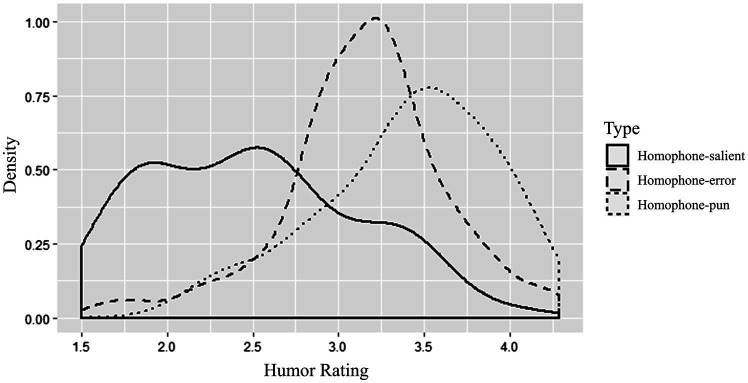
Density plot of humor ratings for each sentence type.

Outlier trials that indicated insufficient processing were eliminated (affecting 2.08% of data): trials where less than 2 (out of the total 5) interest areas were visited, trials with the total reading time less than 1,000 ms or greater than 2.5 SDs. The mean rating score for the experimental sentences was 2.84 (*SD* = 0.42), indicating the experimental materials did not skew toward the humorous sentences. Figure 2 displays the probability distribution of rating scores for each sentence type.

Visual inspection of the density plot reveals that the participants rated around two-thirds of the homophone-pun sentences as funny (*M* > 3). In contrast, most homophone-salient sentences were judged as not funny (*M* < 3). As for the homophone-error sentences, most ratings were around the score of 3, suggesting uncertainty of the participants.

Humor rating scores for the three types of sentences were then analyzed with linear mixed-effect models (Baayen et al., 2008) using R (R Development Core Team, 2019) and the lme4 package (Bates et al., 2015). This approach is more advantageous than traditional analyses (e.g., *t*-test or ANOVA) because it can simultaneously account for separate sources of error variance associated with participants and stimuli in the same statistical model.

In the analysis, sentence *Type* (homophone-pun, homophone-error, and homophone-salient) was defined as the fixed effect and *Participant* and trial *Item* as the random factors. Following the recommendation of Barr et al. (2013), we started the model with a maximum random effect structure, including a random intercept and slope for each participant and trial item. If the model failed to converge, the by-item random slope was dropped first, then the by-participant random slope if necessary. Model comparison was performed using the ANOVA function to select the better model. The following formula was used: lme4 [Rating ~ Type + (1 + Type|Participant) + (1 + Type|Item)]. Following convention, *t* values greater than 2 were treated as significant. This is because the *t*-statistic generally corresponds to the *z*-statistic, considering the small number of fixed and random effects and the large number of observations estimated.

According to the analysis, the participants rated homophone puns as significantly funnier (*M* = 3.39, *SD* = 1.07) than both the homophone-error condition (*M* = 3.13, *SD* = 1.07, *t* = 4.58) and the homophone-salient condition (*M* = 2.49, *SD* = 1.12, *t* = 12.30). Interestingly, the participants also rated the homophone-error sentences as funnier than the homophone-salient sentences (*M* = 3.13 vs. *M* = 2.49).

Since the only difference between the pun sentences and the homophone-error sentences lies in the semantic relatedness between the homophone and the critical context word, we made another analysis using both sentence *Type* and *Semantic Relatedness* as the predictors. According to the analysis, the effect of sentence type was no longer significant (*β* = 0.06, *SE* = 0.08, *t* = 0.77), while the effect of semantic relatedness was significant (*β* = 0.11, *SE* = 0.43, *t* = 2.67), suggesting that *Semantic Relatedness* was more predictive of the rating scores. Specifically, the more the critical context word was semantically related to the less salient homophone, the funnier the participants would rate the sentence, regardless of sentence type.

#### Sentence Reading Time

Sentence reading time was defined as the period from the presentation of a sentence to the moment when the participant pressed the space bar on the keyboard. Statistical analysis was conducted using the same procedure as the funniness rating analysis. Since visual examination of the distribution of the residuals strongly suggested a log-transformation (log10) to meet the LMM assumptions, the reading times were log-transformed for estimation (Holmqvist et al., 2011). According to the analysis, the pun sentences (*M* = 3,678 ms, *SE* = 42 ms, *t* = −3.76) was read significantly faster than the homophone-salient sentences (*M* = 3,867 ms, *SE* = 42 ms), while the homophone-error sentences (*M* = 4,358 ms, *SE* = 48 ms, *t* = 7.87) was read significantly more slowly than the homophone-salient sentences.

### Interest-Area Analysis

Besides the trials eliminated in the rating analysis, fixation shorter than 80 ms or longer than 1,200 ms were also excluded from further analysis (affecting 5.8% of data). Three regions of interest (ROIs) were set up for fixation analysis, namely the critical context word area (ROI_1_), the homophone area (ROI_2_), and the spill-over area (ROI_3_). These interest areas were set up for different purposes: the ROI_1_ to examine the context effect, the ROI_2_ to investigate the processing difficulty of the homophones, and the ROI_3_ to capture possible spill-over effects after reading the homophones. See also Table 1 for an illustration of the setup for different ROIs.

The following eye-movement measures are reported: First fixation duration (FFD), Gaze duration (GD), Total duration (TD), Regression-In proportion (Reg-In), and Regression-Out proportion (Reg-Out). Among those measures, FFD and GD are sensitive to early lexical processing, and TD is both the early lexical processing and the semantic integration processes (Rayner et al., 2004). The regression-based measures can reflect the extra cognitive effort, especially when readers experience integration difficulties and have to regress onto previous regions for more clues. Mean fixation times and the standard error for each measure are reported in Table 3 for the homophone-pun and the homophone-salient sentences; in Table 4 for comparison between the homophone-pun and homophone-error sentences. Eye movement measures were defined as dependent variables and subjected to a series of linear mixed-effects models as in the sentence-level analyses.

**Table 3:** Fixation data of different ROIs in homophone-pun and homophone-salient sentences.

	ROI_1_		ROI_2_		ROI_3_	
	Homophone-pun	Homophone-salient	Homophone-pun	Homophone-salient	Homophone-pun	Homophone-salient
FFD (ms)	215 (3)	212 (2)	275 (3)	251 (3)	241 (3)	235 (3)
GD (ms)	251 (4)	254 (5)	383 (7)	302 (4)	356 (8)	351 (7)
TD (ms)	358 (8)	439 (9)	675 (11)	541 (10)	522 (13)	508 (13)
Reg-In (%)	37.5 (1)	43.8 (1)	39.1 (1)	33.5 (1)	NA	NA
Reg-Out (%)	22.4 (1)	24.9 (1)	29.7 (1)	31.2 (0.01)	84.8 (1)	91.1 (1)

SEs are reported in parentheses.

**Table 4:** Fixation data of different ROIs in homophone-pun and homophone-error sentences.

	ROI_1_		ROI_2_		ROI_3_	
	Homophone-pun	Homophone-error	Homophone-pun	Homophone-error	Homophone-pun	Homophone-error
FFD (ms)	215 (3)	216 (3)	275 (3)	279 (4)	241 (3)	240 (3)
GD (ms)	251 (4)	259 (5)	383 (7)	402 (8)	356 (8)	345 (7)
TD (ms)	358 (8)	472 (10)	675 (11)	823 (14)	522 (13)	602 (16)
Reg-In (%)	37.5 (1)	43.5 (1)	39.1 (1)	47.7 (1)	NA	NA
Reg-Out (%)	22.4 (1)	23.8 (1)	29.7 (1)	32.4 (1)	84.8 (1)	91.2 (1)

SEs are reported in parentheses.

#### The Homophone-Pun vs. the Homophone-Salient Sentences

For the critical context word (ROI_1_), early measures reveal no significant difference between the two conditions (in FFD, *β* = 0.01, *SE* = 0.01, *t* = 1.03; in GD, *β* = 0.00, *SE* = 0.01, *t* = 0.04), which was as expected since the same critical context word were used. Surprisingly, the critical context words were read significantly faster in the pun sentences than in the homophone-salient sentences in TD (*β* = −0.06, *SE* = 0.01, *t* = −5.144). Analysis on the Regression-In data further proved this pattern (*β* = −0.59, *SE* = 0.13, z = −4.48, *p* < 0.001), suggesting that the participants were more likely to look back at the context word again in the homophone-salient sentences.

For the homophone region (ROI_2_), both the early and the late measures indicated that homophones in the pun condition were read significantly more slowly than their salient homophone mates in the homophone-salient sentences (in FFD, *β* = 0.04, *SE* = 0.01, *t* = 4.83; in GD, *β* = 0.08, *SE* = 0.01, *t* = 7.90; and in TD, *β* = 0.10, *SE* = 0.02, *t* = 6.41). Reg-In analysis also revealed that the participants were more likely to look back at the homophones in the pun condition, implying extra effort were needed to process the pun word (*β* = 0.37, *SE* = 0.11, *t* = 3.46, *p* < 0.001).

As mentioned earlier, ROI_3_ was set up to capture the possible spill-over effects. Analyses of the FFD, GD, and TD showed no difference (*t*s < 2) between the two conditions. However, the analysis on the Reg-Out revealed that the participants would look back less when reading the pun sentences (*β* = −0.49, *SE* = 0.17, *z* = −2.913.46, *p* = 0.004), consistent with the finding in ROI_1_; namely, the participants regress less onto the context word in the homophone-pun condition.

#### The Homophone-Pun vs. the Homophone-Error Sentences

For the critical context word (ROI_1_), early measures revealed no significant difference between the homophone-pun and homophone-error sentences (in FFD, *β* = −0.003, *SE* = 0.007, *t* = −0.473; in GD, *β* = −0.005, *SE* = 0.010, *t* = −0.494), an indication that the context words in both types of sentences were matched properly in lexical properties. However, the critical context words were read significantly faster in the homophone-pun sentences than in the homophone-error sentences in TD (*β* = −0.082, *SE* = 0.015, *t* = −5.455). This pattern has been further confirmed by the analysis of the Reg-In data (*β* = −0.320, *SE* = 0.134, *z* = −2.399, *p* = 0.016), which indicates that the participants were more likely to re-examine the context word in the homophone-error sentences.

For the homophone area (ROI_2_), early measures revealed no significant difference between the homophone puns and homophone-error sentences (in FFD, *β* = 0.001, *SE* = 0.006, *t* = −0.187; in GD, *β* = −0.010, *SE* = 0.009, *t* = −1.12), but significant difference in TD (*β* = −0.070, *SE* = 0.011, *t* = −6.53). Reg-In analysis showed that the participants were more likely to recheck the homophones in the homophone-error sentences, suggesting extra effort for the computation of the homophone meaning (*β* = −0.431, *SE* = 0.082, *z* = −5.240, *p* < 0.001).

In the spill-over region (ROI_3_), the analyses on FFD and GD revealed no difference (*t*s < 2) between the two types of sentences. However, the TD analysis revealed that this area was read significantly faster in homophone puns (*β* = −0.052, *SE* = 0.010, *t* = −5.035), suggesting less difficulty in integrating the homophone into the pun context. This pattern has been further supported from analysis on the Reg-Out proportion (*β* = −0.721, *SE* = 0.187, *z* = −3.859, *p* < 0.001), namely the participants regress less onto the previous regions in the homophone-pun sentences.

## Discussion

The current study investigated how Chinese readers dynamically utilize contextual information to appreciate homophone puns. Both humor rating and eye movement results from the homophone-pun and homophone-salient sentences indicated that visual presentation of the less salient homophones led to a greater humor experience rather than the other way around. This finding was consistent with the prediction of the graded salience hypothesis on reading homophone puns (Giora, 1997) and was also resonant with the salience effect reported in previous studies on homograph puns (Coulson and Severens, 2007; Mchugh and Buchanan, 2016; Koleva et al., 2019; Zheng et al., 2020). In addition, the strength of contextual support for the less salient homophone, quantified in the current experiment by the semantic relatedness between the critical context noun and the homophone, was found predictive of the humor ratings in both the homophone-pun and homophone-error conditions.

A comparison of the rating scores between the homophone-salient sentences and the homophone puns reveals the answer to the first research question, i.e., visual presentation of the less salient homophone rather than its salient homophone mate elicited higher humor rating scores. To put it differently, readers were more likely to take a sentence as a pun when the less salient homophone was visually supported in a potential pun context. Current results, therefore, lent support to the prediction of the graded salience hypothesis. As Giora (2003) noted “Language users are sensitive to degrees of salience. No wonder puns tend to spell out the less salient meaning of ambiguous words or expressions, trusting the lexical processor to activate the more salient meaning on its own accord and make the interplay between these meanings possible.” Additionally, the present study extends the current understanding of the salience effect reported in previous research on homograph puns (Coulson and Severens, 2007; Koleva et al., 2019; Wang and Zheng, 2019): although the less salient meaning can be accessed first in reading homophone puns, it is still the salient meaning that is more recoverable even without explicit visual cues or contextual support.

This finding was consistent with the reverse reading pattern in the homophone region (ROI_2_) and the spill-over region (ROI_3_) between the homophone-salient and homophone-pun conditions. As expected, the early (FFD, GD) and late eye-movement measures (TD) in ROI_2_ indicated that the salient homophone was processed faster and integrated more easily into the context than the less salient homophone when presented visually. Therefore, it was natural to assume that the readers would regress less in ROI_3_ when reading the homophone-salient sentence. Previous research also showed that readers would regress less at the sentential-final position when reading non-humorous controls compared to jokes (Coulson et al., 2006). However, an opposite pattern was observed in ROI_3_. According to the Reg-Out data, readers were more likely to look back in the homophone-salient sentences, indicating the meaning of the less salient homophone was then partially activated through retrieved phonological information. Recall that the homophone-salient sentences shared exactly the same context with the homophone puns and differed only in the visually-presented homophones. The activation of the less salient homophone could have led the participants to reexamine the critical context word region (hence, the higher regression-in proportion in ROI_1_ and the longer sentence reading times) before it was finally disconfirmed by the orthographic form of the salient homophone. The suppression of the less salient homophone meaning, then, could have led to the much lower rating scores for the homophone-salient sentences. On the other hand, once the participants figured out the less salient homophone, the recovery and integration of the salient homophone meaning seemed to become much smoother. It should be noted that the task requirements may have also contributed to this finding. In the current experiment, the participants were asked to rate the funniness of each sentence that they had read. This instruction might have also promoted them to regress more in the unfunny homophone-salient sentences. In contrast, when reading the homophone-pun sentences, they were more likely to move on right away once they had got the pun.

Despite the topological difference between Chinese and English, the semantic relatedness between the critical context noun and the less salient homophone was also found predictive of the humor ratings toward the homophone-pun and homophone-error sentences, consistent with the findings in English homophone puns (Jared and Bainbridge, 2017). Specifically, the higher the semantic relatedness between the critical context noun and the less salient homophone is, the higher humor scores the participants would give. Therefore, this measure seems a robust predictor, which punsters could rely on when creating homophone puns. From a more theoretical perspective, this measure could have indexed the extra cognitive effort taken by the readers to search for the optimal relevance between the homophone meanings and the pun context, as proposed by the relevance theory (Sperber and Wilson, 1986; see also Yus, 2003).

Current findings also shed some light on the unique features of a pun context. There is no denying that context usually serves as a crucial factor for ambiguity resolution. Swinney (1979) demonstrated in his classical study that both meanings of an ambiguous homograph are available, such as *bug* (insect; hidden microphone), but the listeners can quickly select the most appropriate meaning based on the context. On the other hand, what makes a pun intriguing is that both meanings can be accessed and integrated into the context. So what is so unique about a pun context that makes a dual meaning interpretation possible?

Lower funniness scores in the homophone-error sentences than the homophone puns suggest that a pun context is needed to directly support the less salient meaning. In the homophone-error sentences, we replaced the critical context noun supporting the less salient homophone with an unrelated word. As suggested by the longer TD on the homophones (ROI_2_), it was more effortful for the participants to figure out the meaning of the less salient homophone in this condition. Additionally, even after the participants figured out the meaning of the less salient homophones, they also had greater difficulty integrating this meaning into the context, indicated by the higher Reg-out from the spill-over region (ROI_3_). It is interesting to note that the readers probably had made a sensible interpretation out of some of the homophone-error sentences by resorting to the meaning of the unpresented salient homophone through shared phonology, as suggested by the humor ratings (*M* = 3.13) and sentence readability ratings (*M* = 3.44). Although this dual-meaning retrieval process in the homophone-error condition may share some similarities with the pun condition, the critical difference lies in that only the salient homophone meaning is compatible with the context in the homophone-error condition. Meanwhile, the less salient homophone meaning would be suppressed without support from the context (Gernsbacher and Faust, 1991), hence the lower humor rating scores.

On the other hand, the pun context is not needed to favor the salient meaning explicitly. In the homophone-salient condition, even though the salient homophones were not directly supported by the critical context nouns (semantic relatedness: *M* = 2.35), they were still read much faster than the less salient homophones (*M* = 3.69) in the homophone puns in terms of FFD, GD, and TD, indicating an advantage of the salient homophones during the meaning-access process. Nevertheless, the salient meanings still need to be semantically compatible with the overall sentence context, as can be seen from the readability score of the homophone-salient sentences (*M* = 3.81); otherwise, the retrieved salient meanings will also be discarded during the ensuing semantic integration process, leaving the dual-meaning juxtaposition impossible.

The comparatively high rating scores of the homophone-error sentences (*M* = 3.13) in the current study differed from those reported by Jared and Bainbridge (2017). In their study, the average funniness rating scores of the homophone-error sentences are much lower (*M* = 1.6). Although this difference can be partially attributed to the material difference, it deserves further explanations. One possible explanation for this discrepancy is the orthographic differences between English and Chinese. To be more specific, homophones used in the study of Jared and Bainbridge (2017) were more likely to be misread as their unpresented homophone mates, especially when the latter was more salient. This is because in alphabetic languages like English, homophonic pairs often share considerable visual similarities. In their example, *The butcher was very glad we could meat up*, the homophone pair (*meat-meet*) differed in just one letter. The possibility of misreading can be further increased when the unpresented homophone mate is more salient or frequent. Niznikiewicz and Squires (1996) also raised the similar idea that an incorrect homophone in a sentential context (e.g., *Jane will come by plain*) will be likely processed as if it were its correct homophone (*plane*). Indeed, it is well established that phonological information is activated very early in alphabetic scripts (Rayner et al., 1998; Morris and Folk, 2000). On the other hand, the Chinese writing system is often considered a deep orthography, whose orthography-phonology mapping is more inconsistent. Chinese characters with the same pronunciation may look completely different, as in the first character of the homophone pair *男题-难题*. Besides, some studies claimed that the phonological mediation could be bypassed in skilled Chinese readers, and semantic information could be accessed directly from the orthographic route (Zhou and Marslen-Wilson, 1999; Chen and Shu, 2001; Zhou et al., 2018). Therefore, it is highly unlikely for participants in the current study to mistake the homophones as their unpresented homophone mates through shared phonology. Instead, they were more likely to treat the less salient homophones, many being temporarily made-up compound words, as a kind of novel expression, hence the higher rating score (Giora et al., 2004).

Two limitations of the current study have to be noted. Firstly, the experiment task could have resulted in a more strategic reading pattern different from normal reading. In the present experiment, participants were asked to give a funniness rating score after finishing reading each sentence. Some participants, especially more careful readers, could spend more time reading the nonhumorous sentences. This could partially explain why participants regressed more to the context nouns in the homophone-salient condition than in the homophone-pun condition. Future studies could use a different task, such as the readability rating task, to further test the robustness of current findings. Secondly, although the eye-tracking technique was used in the experiment and served well for our research objectives, the present study provides little neurological insights into the humor appreciation process of homophone puns. For example, although the extra cognitive effort made by the readers could have led to the humor experience in reading homophone puns, little is known about how these two processes are related neurologically. In the field of humor comprehension, some researchers have already investigated the neurological basis for joke comprehension using techniques such as ERPs (Coulson and Kutas, 2001; Chan, 2016) and fMRI (Chan et al., 2012; Chan, 2016). Although punning is an essential type of verbal humor, till now, little such research on homophone puns can be found in the literature. As a result, further investigation from a neurological or physiological perspective is promising in shedding more light on the processing of homophone puns.

## Data Availability Statement

The raw data supporting the conclusions of this article will be made available by the authors, without undue reservation.

## Ethics Statement

The studies involving human participants were reviewed and approved by Research Ethics Board of Zhejiang University. The patients/participants provided their written informed consent to participate in this study.

## Author Contributions

WZ and XW conceived and designed the experiments and revised the manuscript. WZ performed the experiments and analyzed the data and wrote the manuscript. All authors contributed to the article and approved the submitted version.

## Funding

This study is supported by the Major Project of National Social Science Foundation of China (14ZDB155).

## Conflict of Interest

The authors declare that the research was conducted in the absence of any commercial or financial relationships that could be construed as a potential conflict of interest.

## Publisher’s Note

All claims expressed in this article are solely those of the authors and do not necessarily represent those of their affiliated organizations, or those of the publisher, the editors and the reviewers. Any product that may be evaluated in this article, or claim that may be made by its manufacturer, is not guaranteed or endorsed by the publisher.

## Footnotes

^1^The N400 component is a centroparietal negative brain potential, which is known to be sensitive to semantic violations or anomaly (Kutas and Hillyard, 1980). It is often used as an index for semantic priming, with a smaller N400 component indicating a larger priming effect (Kutas and Federmeier, 2011).
